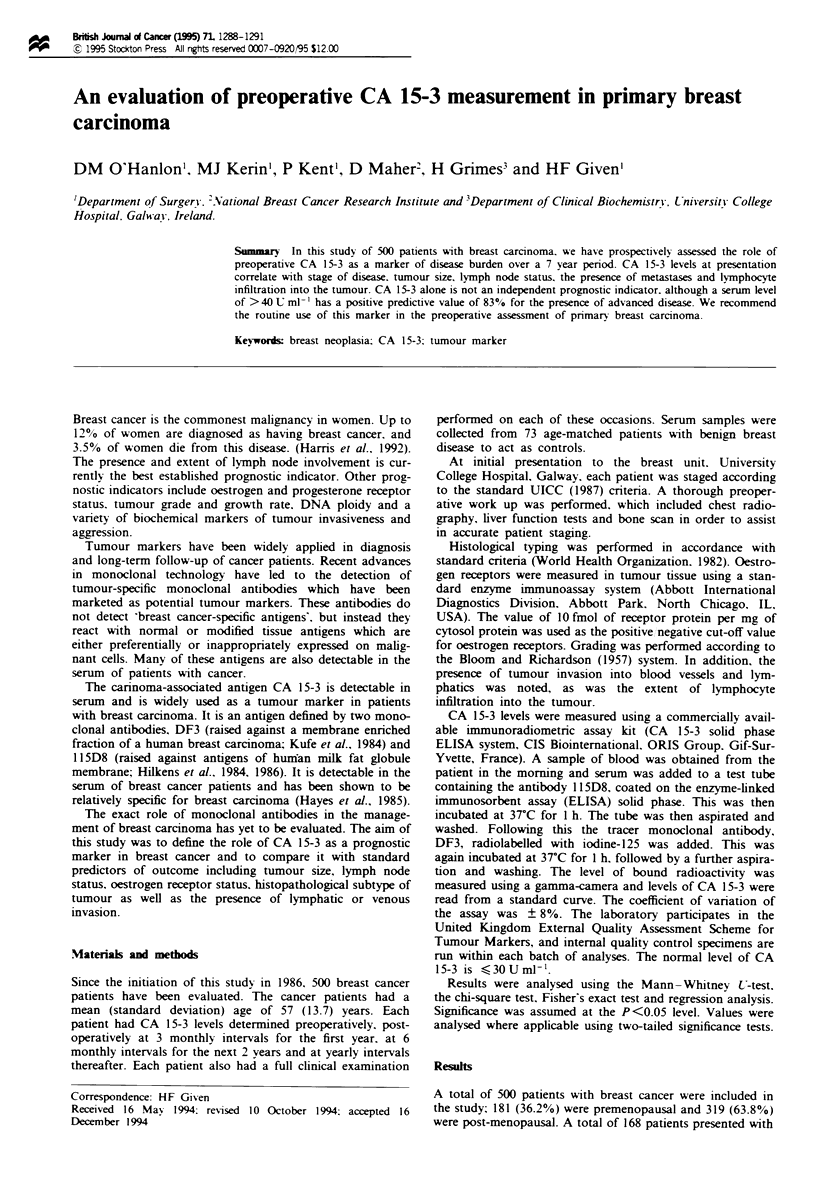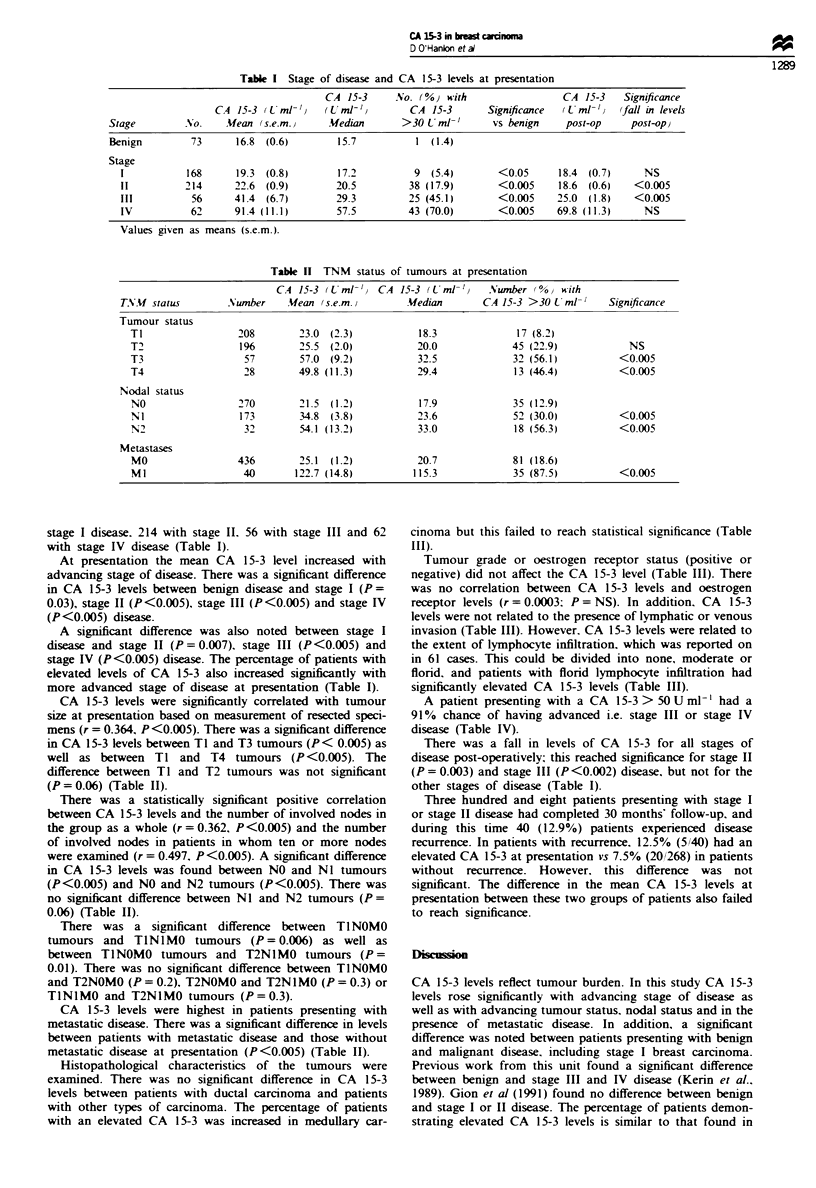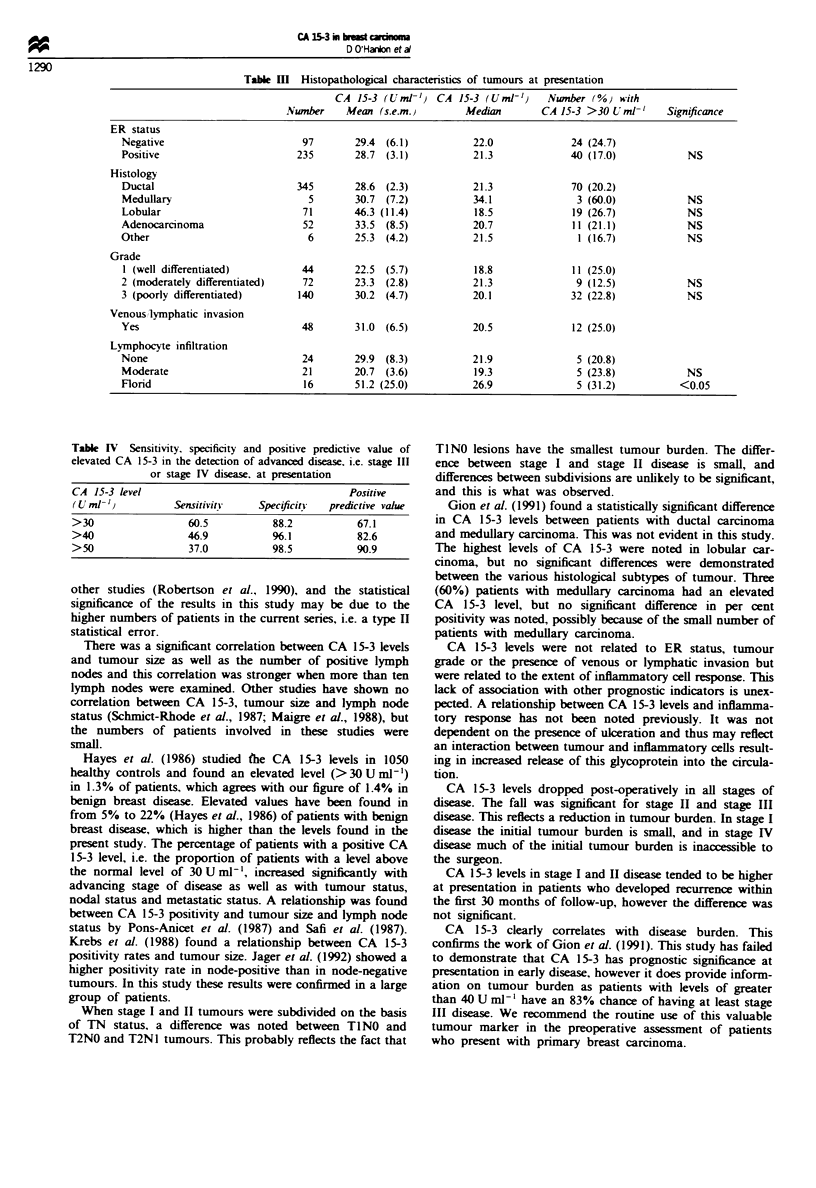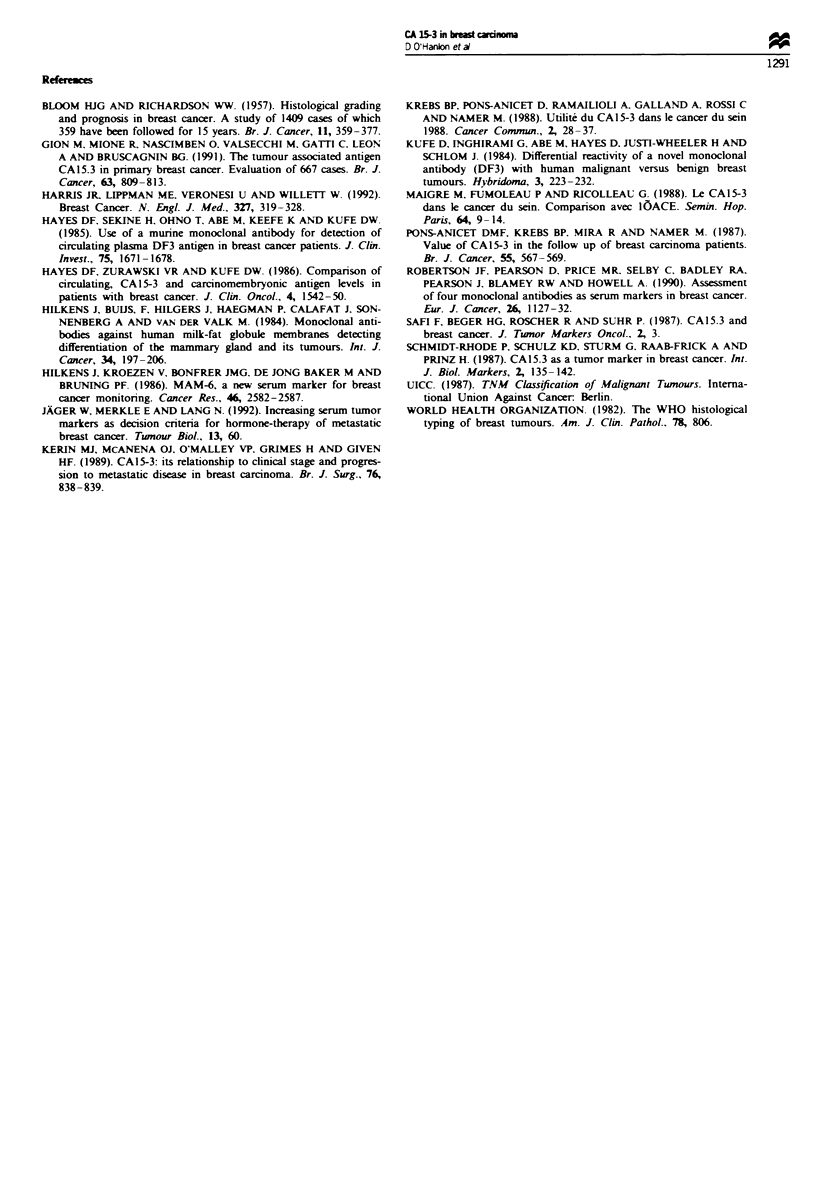# An evaluation of preoperative CA 15-3 measurement in primary breast carcinoma.

**DOI:** 10.1038/bjc.1995.249

**Published:** 1995-06

**Authors:** D. M. O'Hanlon, M. J. Kerin, P. Kent, D. Maher, H. Grimes, H. F. Given

**Affiliations:** Department of Surgery, University College Hospital, Galway, Ireland.

## Abstract

In this study of 500 patients with breast carcinoma, we have prospectively assessed the role of preoperative CA 15-3 as a marker of disease burden over a 7 year period. CA 15-3 levels at presentation correlate with stage of disease, tumour size, lymph node status, the presence of metastases and lymphocyte infiltration into the tumour. CA 15-3 alone is not an independent prognostic indicator, although a serum level of > 40 U ml-1 has a positive predictive value of 83% for the presence of advanced disease. We recommend the routine use of this marker in the preoperative assessment of primary breast carcinoma.


© 1995 Stockton Press All rights reserved 0007-0920/95 $12.00

### An evaluation of preoperative CA 15-3 measurement in primary breast carcinoma

DM O'Hanlon[1], MJ Kerin[1], P Kent[1], D Maher[2], H Grimes[3] and HF Given[1]

[1]*Department of Surgery*, [2]*National Breast Cancer Research Institute and* [3]*Department of Clinical Biochemistry, University College Hospital, Galway, Ireland.*

**Summary** In this study of 500 patients with breast carcinoma, we have prospectively assessed the role of preoperative CA 15-3 as a marker of disease burden over a 7 year period. CA 15-3 levels at presentation correlate with stage of disease, tumour size, lymph node status, the presence of metastases and lymphocyte infiltration into the tumour. CA 15-3 alone is not an independent prognostic indicator, although a serum level of $>40\ U\ ml^{-1}$ has a positive predictive value of 83% for the presence of advanced disease. We recommend the routine use of this marker in the preoperative assessment of primary breast carcinoma.

**Keywords:** breast neoplasia; CA 15-3; tumour marker

Breast cancer is the commonest malignancy in women. Up to 12% of women are diagnosed as having breast cancer, and 3.5% of women die from this disease. (Harris *et al*., 1992). The presence and extent of lymph node involvement is currently the best established prognostic indicator. Other prognostic indicators include oestrogen and progesterone receptor status, tumour grade and growth rate, DNA ploidy and a variety of biochemical markers of tumour invasiveness and aggression.

Tumour markers have been widely applied in diagnosis and long-term follow-up of cancer patients. Recent advances in monoclonal technology have led to the detection of tumour-specific monoclonal antibodies which have been marketed as potential tumour markers. These antibodies do not detect 'breast cancer-specific antigens', but instead they react with normal or modified tissue antigens which are either preferentially or inappropriately expressed on malignant cells. Many of these antigens are also detectable in the serum of patients with cancer.

The carinoma-associated antigen CA 15-3 is detectable in serum and is widely used as a tumour marker in patients with breast carcinoma. It is an antigen defined by two monoclonal antibodies, DF3 (raised against a membrane enriched fraction of a human breast carcinoma; Kufe *et al*., 1984) and 115D8 (raised against antigens of human milk fat globule membrane; Hilkens *et al*., 1984, 1986). It is detectable in the serum of breast cancer patients and has been shown to be relatively specific for breast carcinoma (Hayes *et al*., 1985).

The exact role of monoclonal antibodies in the management of breast carcinoma has yet to be evaluated. The aim of this study was to define the role of CA 15-3 as a prognostic marker in breast cancer and to compare it with standard predictors of outcome including tumour size, lymph node status, oestrogen receptor status, histopathological subtype of tumour as well as the presence of lymphatic or venous invasion.

### Materials and methods

Since the initiation of this study in 1986, 500 breast cancer patients have been evaluated. The cancer patients had a mean (standard deviation) age of 57 (13.7) years. Each patient had CA 15-3 levels determined preoperatively, postoperatively at 3 monthly intervals for the first year, at 6 monthly intervals for the next 2 years and at yearly intervals thereafter. Each patient also had a full clinical examination performed on each of these occasions. Serum samples were collected from 73 age-matched patients with benign breast disease to act as controls.

At initial presentation to the breast unit, University College Hospital, Galway, each patient was staged according to the standard UICC (1987) criteria. A thorough preoperative work up was performed, which included chest radiography, liver function tests and bone scan in order to assist in accurate patient staging.

Histological typing was performed in accordance with standard criteria (World Health Organization, 1982). Oestrogen receptors were measured in tumour tissue using a standard enzyme immunoassay system (Abbott International Diagnostics Division, Abbott Park, North Chicago, IL, USA). The value of 10 fmol of receptor protein per mg of cytosol protein was used as the positive/negative cut-off value for oestrogen receptors. Grading was performed according to the Bloom and Richardson (1957) system. In addition, the presence of tumour invasion into blood vessels and lymphatics was noted, as was the extent of lymphocyte infiltration into the tumour.

CA 15-3 levels were measured using a commercially available immunoradiometric assay kit (CA 15-3 solid phase ELISA system, CIS Biointernational, ORIS Group, Gif-Sur-Yvette, France). A sample of blood was obtained from the patient in the morning and serum was added to a test tube containing the antibody 115D8, coated on the enzyme-linked immunosorbent assay (ELISA) solid phase. This was then incubated at 37°C for 1 h. The tube was then aspirated and washed. Following this the tracer monoclonal antibody, DF3, radiolabelled with iodine-125 was added. This was again incubated at 37°C for 1 h, followed by a further aspiration and washing. The level of bound radioactivity was measured using a gamma-camera and levels of CA 15-3 were read from a standard curve. The coefficient of variation of the assay was ± 8%. The laboratory participates in the United Kingdom External Quality Assessment Scheme for Tumour Markers, and internal quality control specimens are run within each batch of analyses. The normal level of CA 15-3 is $\leqslant 30\ U\ ml^{-1}$.

Results were analysed using the Mann-Whitney *U*-test, the chi-square test, Fisher's exact test and regression analysis. Significance was assumed at the $P<0.05$ level. Values were analysed where applicable using two-tailed significance tests.

### Results

A total of 500 patients with breast cancer were included in the study; 181 (36.2%) were premenopausal and 319 (63.8%) were post-menopausal. A total of 168 patients presented with

Correspondence: HF Given
Received 16 May 1994; revised 10 October 1994; accepted 16 December 1994

**Table I** Stage of disease and CA 15-3 levels at presentation

| Stage | No. | CA 15-3 ($U ml^{-1}$) Mean (s.e.m.) | CA 15-3 ($U ml^{-1}$) Median | No. (%) with CA 15-3 >30 $U ml^{-1}$ | Significance vs benign | CA 15-3 ($U ml^{-1}$) post-op | Significance (fall in levels post-op) |
|---|---|---|---|---|---|---|---|
| Benign | 73 | 16.8 (0.6) | 15.7 | 1 (1.4) | | | |
| Stage | | | | | | | |
| I | 168 | 19.3 (0.8) | 17.2 | 9 (5.4) | <0.05 | 18.4 (0.7) | NS |
| II | 214 | 22.6 (0.9) | 20.5 | 38 (17.9) | <0.005 | 18.6 (0.6) | <0.005 |
| III | 56 | 41.4 (6.7) | 29.3 | 25 (45.1) | <0.005 | 25.0 (1.8) | <0.005 |
| IV | 62 | 91.4 (11.1) | 57.5 | 43 (70.0) | <0.005 | 69.8 (11.3) | NS |

Values given as means (s.e.m.).

**Table II** TNM status of tumours at presentation

| TNM status | Number | CA 15-3 ($U ml^{-1}$) Mean (s.e.m.) | CA 15-3 ($U ml^{-1}$) Median | Number (%) with CA 15-3 >30 $U ml^{-1}$ | Significance |
|---|---|---|---|---|---|
| Tumour status | | | | | |
| T1 | 208 | 23.0 (2.3) | 18.3 | 17 (8.2) | |
| T2 | 196 | 25.5 (2.0) | 20.0 | 45 (22.9) | NS |
| T3 | 57 | 57.0 (9.2) | 32.5 | 32 (56.1) | <0.005 |
| T4 | 28 | 49.8 (11.3) | 29.4 | 13 (46.4) | <0.005 |
| Nodal status | | | | | |
| N0 | 270 | 21.5 (1.2) | 17.9 | 35 (12.9) | |
| N1 | 173 | 34.8 (3.8) | 23.6 | 52 (30.0) | <0.005 |
| N2 | 32 | 54.1 (13.2) | 33.0 | 18 (56.3) | <0.005 |
| Metastases | | | | | |
| M0 | 436 | 25.1 (1.2) | 20.7 | 81 (18.6) | |
| M1 | 40 | 122.7 (14.8) | 115.3 | 35 (87.5) | <0.005 |

stage I disease, 214 with stage II, 56 with stage III and 62 with stage IV disease (Table I).

At presentation the mean CA 15-3 level increased with advancing stage of disease. There was a significant difference in CA 15-3 levels between benign disease and stage I ($P=0.03$), stage II ($P<0.005$), stage III ($P<0.005$) and stage IV ($P<0.005$) disease.

A significant difference was also noted between stage I disease and stage II ($P=0.007$), stage III ($P<0.005$) and stage IV ($P<0.005$) disease. The percentage of patients with elevated levels of CA 15-3 also increased significantly with more advanced stage of disease at presentation (Table I).

CA 15-3 levels were significantly correlated with tumour size at presentation based on measurement of resected specimens ($r=0.364$, $P<0.005$). There was a significant difference in CA 15-3 levels between T1 and T3 tumours ($P<0.005$) as well as between T1 and T4 tumours ($P<0.005$). The difference between T1 and T2 tumours was not significant ($P=0.06$) (Table II).

There was a statistically significant positive correlation between CA 15-3 levels and the number of involved nodes in the group as a whole ($r=0.362$, $P<0.005$) and the number of involved nodes in patients in whom ten or more nodes were examined ($r=0.497$, $P<0.005$). A significant difference in CA 15-3 levels was found between N0 and N1 tumours ($P<0.005$) and N0 and N2 tumours ($P<0.005$). There was no significant difference between N1 and N2 tumours ($P=0.06$) (Table II).

There was a significant difference between T1N0M0 tumours and T1N1M0 tumours ($P=0.006$) as well as between T1N0M0 tumours and T2N1M0 tumours ($P=0.01$). There was no significant difference between T1N0M0 and T2N0M0 ($P=0.2$), T2N0M0 and T2N1M0 ($P=0.3$) or T1N1M0 and T2N1M0 tumours ($P=0.3$).

CA 15-3 levels were highest in patients presenting with metastatic disease. There was a significant difference in levels between patients with metastatic disease and those without metastatic disease at presentation ($P<0.005$) (Table II).

Histopathological characteristics of the tumours were examined. There was no significant difference in CA 15-3 levels between patients with ductal carcinoma and patients with other types of carcinoma. The percentage of patients with an elevated CA 15-3 was increased in medullary carcinoma but this failed to reach statistical significance (Table III).

Tumour grade or oestrogen receptor status (positive or negative) did not affect the CA 15-3 level (Table III). There was no correlation between CA 15-3 levels and oestrogen receptor levels ($r=0.0003$; $P=$ NS). In addition, CA 15-3 levels were not related to the presence of lymphatic or venous invasion (Table III). However, CA 15-3 levels were related to the extent of lymphocyte infiltration, which was reported on in 61 cases. This could be divided into none, moderate or florid, and patients with florid lymphocyte infiltration had significantly elevated CA 15-3 levels (Table III).

A patient presenting with a CA 15-3 > 50 U $ml^{-1}$ had a 91% chance of having advanced i.e. stage III or stage IV disease (Table IV).

There was a fall in levels of CA 15-3 for all stages of disease post-operatively; this reached significance for stage II ($P=0.003$) and stage III ($P<0.002$) disease, but not for the other stages of disease (Table I).

Three hundred and eight patients presenting with stage I or stage II disease had completed 30 months' follow-up, and during this time 40 (12.9%) patients experienced disease recurrence. In patients with recurrence, 12.5% (5/40) had an elevated CA 15-3 at presentation *vs* 7.5% (20/268) in patients without recurrence. However, this difference was not significant. The difference in the mean CA 15-3 levels at presentation between these two groups of patients also failed to reach significance.

### Discussion

CA 15-3 levels reflect tumour burden. In this study CA 15-3 levels rose significantly with advancing stage of disease as well as with advancing tumour status, nodal status and in the presence of metastatic disease. In addition, a significant difference was noted between patients presenting with benign and malignant disease, including stage I breast carcinoma. Previous work from this unit found a significant difference between benign and stage III and IV disease (Kerin *et al.*, 1989). Gion *et al* (1991) found no difference between benign and stage I or II disease. The percentage of patients demonstrating elevated CA 15-3 levels is similar to that found in

Table III Histopathological characteristics of tumours at presentation

| | Number | CA 15-3 (U $ml^{-1}$) Mean (s.e.m.) | CA 15-3 (U $ml^{-1}$) Median | Number (%) with CA 15-3 >30 U $ml^{-1}$ | Significance |
|---|---|---|---|---|---|
| ER status | | | | | |
| Negative | 97 | 29.4 (6.1) | 22.0 | 24 (24.7) | |
| Positive | 235 | 28.7 (3.1) | 21.3 | 40 (17.0) | NS |
| Histology | | | | | |
| Ductal | 345 | 28.6 (2.3) | 21.3 | 70 (20.2) | |
| Medullary | 5 | 30.7 (7.2) | 34.1 | 3 (60.0) | NS |
| Lobular | 71 | 46.3 (11.4) | 18.5 | 19 (26.7) | NS |
| Adenocarcinoma | 52 | 33.5 (8.5) | 20.7 | 11 (21.1) | NS |
| Other | 6 | 25.3 (4.2) | 21.5 | 1 (16.7) | NS |
| Grade | | | | | |
| 1 (well differentiated) | 44 | 22.5 (5.7) | 18.8 | 11 (25.0) | |
| 2 (moderately differentiated) | 72 | 23.3 (2.8) | 21.3 | 9 (12.5) | NS |
| 3 (poorly differentiated) | 140 | 30.2 (4.7) | 20.1 | 32 (22.8) | NS |
| Venous/lymphatic invasion | | | | | |
| Yes | 48 | 31.0 (6.5) | 20.5 | 12 (25.0) | |
| Lymphocyte infiltration | | | | | |
| None | 24 | 29.9 (8.3) | 21.9 | 5 (20.8) | |
| Moderate | 21 | 20.7 (3.6) | 19.3 | 5 (23.8) | NS |
| Florid | 16 | 51.2 (25.0) | 26.9 | 5 (31.2) | <0.05 |

Table IV Sensitivity, specificity and positive predictive value of elevated CA 15-3 in the detection of advanced disease, i.e. stage III or stage IV disease, at presentation

| CA 15-3 level (U $ml^{-1}$) | Sensitivity | Specificity | Positive predictive value |
|---|---|---|---|
| >30 | 60.5 | 88.2 | 67.1 |
| >40 | 46.9 | 96.1 | 82.6 |
| >50 | 37.0 | 98.5 | 90.9 |

other studies (Robertson et al., 1990), and the statistical significance of the results in this study may be due to the higher numbers of patients in the current series, i.e. a type II statistical error.

There was a significant correlation between CA 15-3 levels and tumour size as well as the number of positive lymph nodes and this correlation was stronger when more than ten lymph nodes were examined. Other studies have shown no correlation between CA 15-3, tumour size and lymph node status (Schmict-Rhode et al., 1987; Maigre et al., 1988), but the numbers of patients involved in these studies were small.

Hayes et al. (1986) studied the CA 15-3 levels in 1050 healthy controls and found an elevated level (>30 U $ml^{-1}$) in 1.3% of patients, which agrees with our figure of 1.4% in benign breast disease. Elevated values have been found in from 5% to 22% (Hayes et al., 1986) of patients with benign breast disease, which is higher than the levels found in the present study. The percentage of patients with a positive CA 15-3 level, i.e. the proportion of patients with a level above the normal level of 30 U $ml^{-1}$, increased significantly with advancing stage of disease as well as with tumour status, nodal status and metastatic status. A relationship was found between CA 15-3 positivity and tumour size and lymph node status by Pons-Anicet et al. (1987) and Safi et al. (1987). Krebs et al. (1988) found a relationship between CA 15-3 positivity rates and tumour size. Jager et al. (1992) showed a higher positivity rate in node-positive than in node-negative tumours. In this study these results were confirmed in a large group of patients.

When stage I and II tumours were subdivided on the basis of TN status, a difference was noted between T1N0 and T2N0 and T2N1 tumours. This probably reflects the fact that T1N0 lesions have the smallest tumour burden. The difference between stage I and stage II disease is small, and differences between subdivisions are unlikely to be significant, and this is what was observed.

Gion et al. (1991) found a statistically significant difference in CA 15-3 levels between patients with ductal carcinoma and medullary carcinoma. This was not evident in this study. The highest levels of CA 15-3 were noted in lobular carcinoma, but no significant differences were demonstrated between the various histological subtypes of tumour. Three (60%) patients with medullary carcinoma had an elevated CA 15-3 level, but no significant difference in per cent positivity was noted, possibly because of the small number of patients with medullary carcinoma.

CA 15-3 levels were not related to ER status, tumour grade or the presence of venous or lymphatic invasion but were related to the extent of inflammatory cell response. This lack of association with other prognostic indicators is unexpected. A relationship between CA 15-3 levels and inflammatory response has not been noted previously. It was not dependent on the presence of ulceration and thus may reflect an interaction between tumour and inflammatory cells resulting in increased release of this glycoprotein into the circulation.

CA 15-3 levels dropped post-operatively in all stages of disease. The fall was significant for stage II and stage III disease. This reflects a reduction in tumour burden. In stage I disease the initial tumour burden is small, and in stage IV disease much of the initial tumour burden is inaccessible to the surgeon.

CA 15-3 levels in stage I and II disease tended to be higher at presentation in patients who developed recurrence within the first 30 months of follow-up, however the difference was not significant.

CA 15-3 clearly correlates with disease burden. This confirms the work of Gion et al. (1991). This study has failed to demonstrate that CA 15-3 has prognostic significance at presentation in early disease, however it does provide information on tumour burden as patients with levels of greater than 40 U $ml^{-1}$ have an 83% chance of having at least stage III disease. We recommend the routine use of this valuable tumour marker in the preoperative assessment of patients who present with primary breast carcinoma.

### References

BLOOM HJG AND RICHARDSON WW. (1957). Histological grading and prognosis in breast cancer. A study of 1409 cases of which 359 have been followed for 15 years. *Br. J. Cancer*, **11**, 359–377.

GION M, MIONE R, NASCIMBEN O, VALSECCHI M, GATTI C, LEON A AND BRUSCAGNIN BG. (1991). The tumour associated antigen CA15.3 in primary breast cancer. Evaluation of 667 cases. *Br. J. Cancer*, **63**, 809–813.

HARRIS JR, LIPPMAN ME, VERONESI U AND WILLETT W. (1992). Breast Cancer. *N. Engl. J. Med.*, **327**, 319–328.

HAYES DF, SEKINE H, OHNO T, ABE M, KEEFE K AND KUFE DW. (1985). Use of a murine monoclonal antibody for detection of circulating plasma DF3 antigen in breast cancer patients. *J. Clin. Invest.*, **75**, 1671–1678.

HAYES DF, ZURAWSKI VR AND KUFE DW. (1986). Comparison of circulating, CA15-3 and carcinomembryonic antigen levels in patients with breast cancer. *J. Clin. Oncol.*, **4**, 1542–50.

HILKENS J, BUIJS, F, HILGERS J, HAEGMAN P, CALAFAT J, SONNENBERG A AND VAN DER VALK M. (1984). Monoclonal antibodies against human milk-fat globule membranes detecting differentiation of the mammary gland and its tumours. *Int. J. Cancer*, **34**, 197–206.

HILKENS J, KROEZEN V, BONFRER JMG, DE JONG BAKER M AND BRUNING PF. (1986). MAM-6, a new serum marker for breast cancer monitoring. *Cancer Res.*, **46**, 2582–2587.

JÄGER W, MERKLE E AND LANG N. (1992). Increasing serum tumor markers as decision criteria for hormone-therapy of metastatic breast cancer. *Tumour Biol.*, **13**, 60.

KERIN MJ, MCANENA OJ, O'MALLEY VP, GRIMES H AND GIVEN HF. (1989). CA15-3: its relationship to clinical stage and progression to metastatic disease in breast carcinoma. *Br. J. Surg.*, **76**, 838–839.

KREBS BP, PONS-ANICET D, RAMAILIOLI A, GALLAND A, ROSSI C AND NAMER M. (1988). Utilité du CA15-3 dans le cancer du sein 1988. *Cancer Commun.*, **2**, 28–37.

KUFE D, INGHIRAMI G, ABE M, HAYES D, JUSTI-WHEELER H AND SCHLOM J. (1984). Differential reactivity of a novel monoclonal antibody (DF3) with human malignant versus benign breast tumours. *Hybridoma*, **3**, 223–232.

MAIGRE M, FUMOLEAU P AND RICOLLEAU G. (1988). Le CA15-3 dans le cancer du sein. Comparison avec lÕACE. *Semin. Hop. Paris*, **64**, 9–14.

PONS-ANICET DMF, KREBS BP, MIRA R AND NAMER M. (1987). Value of CA15-3 in the follow up of breast carcinoma patients. *Br. J. Cancer*, **55**, 567–569.

ROBERTSON JF, PEARSON D, PRICE MR, SELBY C, BADLEY RA, PEARSON J, BLAMEY RW AND HOWELL A. (1990). Assessment of four monoclonal antibodies as serum markers in breast cancer. *Eur. J. Cancer*, **26**, 1127–32.

SAFI F, BEGER HG, ROSCHER R AND SUHR P. (1987). CA15.3 and breast cancer. *J. Tumor Markers Oncol.*, **2**, 3.

SCHMIDT-RHODE P, SCHULZ KD, STURM G, RAAB-FRICK A AND PRINZ H. (1987). CA15.3 as a tumor marker in breast cancer. *Int. J. Biol. Markers*, **2**, 135–142.

UICC. (1987). *TNM Classification of Malignant Tumours*. International Union Against Cancer: Berlin.

WORLD HEALTH ORGANIZATION. (1982). The WHO histological typing of breast tumours. *Am. J. Clin. Pathol.*, **78**, 806.